# Genetic Dissection of Growth Traits in a Unique Chicken Advanced Intercross Line

**DOI:** 10.3389/fgene.2020.00894

**Published:** 2020-09-04

**Authors:** Yuzhe Wang, Lina Bu, Xuemin Cao, Hao Qu, Chunyuan Zhang, Jiangli Ren, Zhuolin Huang, Yiqiang Zhao, Chenglong Luo, Xiaoxiang Hu, Dingming Shu, Ning Li

**Affiliations:** ^1^College of Animal Science and Technology, China Agricultural University, Beijing, China; ^2^State Key Laboratory of Agrobiotechnology, College of Biological Sciences, China Agricultural University, Beijing, China; ^3^State Key Laboratory of Livestock and Poultry Breeding, Guangdong Key Laboratory of Animal Breeding and Nutrition, Institute of Animal Science, Guangdong Academy of Agricultural Sciences, Guangzhou, China

**Keywords:** chicken, advanced intercross line, bone growth, ancestral inference, QTL fine-mapping, genome-wide association study, selective sweep, haplotype association study

## Abstract

The advanced intercross line (AIL) that is created by successive generations of pseudo-random mating after the F_2_ generation is a valuable resource, especially in agricultural livestock and poultry species, because it improves the precision of quantitative trait loci (QTL) mapping compared with traditional association populations by introducing more recombination events. The growth traits of broilers have significant economic value in the chicken industry, and many QTLs affecting growth traits have been identified, especially on chromosomes 1, 4, and 27, albeit with large confidence intervals that potentially contain dozens of genes. To promote a better understanding of the underlying genetic architecture of growth trait differences, specifically body weight and bone development, in this study, we report a nine-generation AIL derived from two divergent outbred lines: High Quality chicken Line A (HQLA) and Huiyang Bearded (HB) chicken. We evaluate the genetic architecture of the F_0_, F_2_, F_8_, and F_9_ generations of AIL and demonstrate that the population of the F_9_ generation sufficiently randomized the founder genomes and has the characteristics of rapid linkage disequilibrium decay, limited allele frequency decline, and abundant nucleotide diversity. This AIL yielded a much narrower QTL than the F_2_ generations, especially the QTL on chromosome 27, which was reduced to 120 Kb. An ancestral haplotype association analysis showed that most of the dominant haplotypes are inherited from HQLA but with fluctuation of the effects between them. We highlight the important role of four candidate genes (*PHOSPHO1*, *IGF2BP1*, *ZNF652*, and *GIP*) in bone growth. We also retrieved a missing QTL from AIL on chromosome 4 by identifying the founder selection signatures, which are explained by the loss of association power that results from rare alleles. Our study provides a reasonable resource for detecting quantitative trait genes and tracking ancestor history and will facilitate our understanding of the genetic mechanisms underlying chicken bone growth.

## Introduction

Identifying key polymorphisms and dissecting the genetic architecture of complex growth traits is of considerable interest in fields like agriculture breeding and evolution. F_2_ crosses between divergent outbred lines are traditionally used to map quantitative trait loci (QTL) in domestic animal and plant populations (Andersson et al., 1994; Perez-Enciso et al., 2001). However, it is not enough to rely on a single generation of meiotic recombination to break up and randomize the parental genomes to finely map causal variants of complex quantitative traits (Flint et al., 2005). Improved strategies, such as the use of larger sample cohorts, the construction of advanced intercross lines (AIL) (Besnier et al., 2011; Parker et al., 2012), nested association mapping population (NAM), and multi-parent advanced generation inter-cross (MAGIC) in animals and plants (Poland et al., 2011; Gatti et al., 2014; Pascual et al., 2015) can increase the precision of quantitative trait loci (QTL) mapping by introducing more recombination events and together provide a series of alternatives to the traditional association mapping of populations.

Advanced intercross lines (AILs) were first introduced by Darvasi and Soller (1995). An AIL is created by successive generations of pseudo-random mating after the F_2_ generation, and recombinations are accumulated continuously between generations and are easier to construct in species with short generation intervals and a high tolerance of inbreeding decline. To date, AIL has been used as a common strategy to improve the mapping resolution for the genome wide association studies (GWASs) of model animals, such as fruit flies (Mackay et al., 2012), mice (Gonzales et al., 2018), chicken (Zan et al., 2017), and *C. elegans* (Doitsidou et al., 2016). The significant advantages of AILs include reducing the QTL confidence interval by 3- to 27-fold and finely splitting the original QTL into two linked QTLs (Besnier et al., 2011; Parker et al., 2014; Arends et al., 2016). However, we should always pay attention to the tradeoff between mapping resolution and statistical power, as the causal allele may become rare with a continuous increase of the inbreeding coefficient in the AIL (Yalcin et al., 2010; Parker et al., 2016).

**FIGURE 1:**
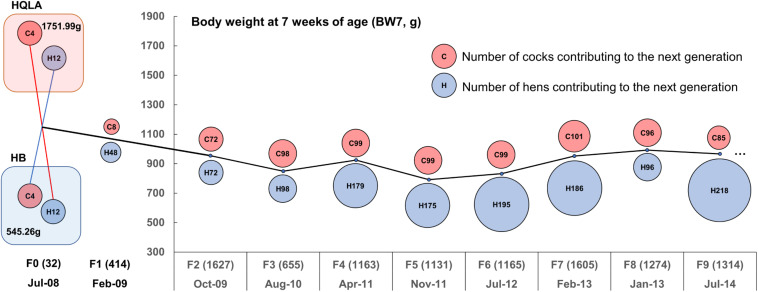
Descriptive statistics for the nine-generation advanced intercross line (AIL) pedigree. The AIL was initiated with 16 High Quality chicken Line A (HQLA) and 16 Huiyang Bearded (HB) chicken in July 2008 with three generations every 2 years on average. The circle size represents the number of individuals contributing to the next generation (red for cocks and blue for hens), and the number in parentheses records the sample size of each generation. The ordinate curve represents the sex-averaged mean body weights at 7 weeks of age.

Bone growth is crucial to poultry production, as skeletal problems are associated with economic benefits and animal welfare issues (Tsudzuki et al., 2007; Kapell et al., 2012). Too long legs give rise to leg problems in high body weight chickens (Deeb and Lamont, 2002). In healthy chicken, shank length (SL) and shank circumference (SC) are the two most commonly used parameters for evaluating bone growth in chickens (Tsudzuki et al., 2007) and are highly correlated with body weight (BW) (Gao et al., 2010). Moreover, shank traits can be measured without slaughter and we can track bone growth of different periods. We previously reported two major QTLs for growth traits located on chicken chromosome 1 (GGA1) and GGA27 via a linkage analysis in the F_2_ generation (Sheng et al., 2013). To promote a better understanding of the underlying genetic architecture for growth trait differences, specifically body weight and bone development, here, we report a nine-generation AIL derived from High Quality chicken Line A (HQLA) × Huiyang Bearded (HB) chicken. Detailed information on HQLA, HB, and AIL is presented in the materials and methods and Figure 1. We employed genotyping-by-sequencing (GBS) SNPs from F_0_, F_8_, and F_9_ (Wang et al., 2017) and Beadchip SNPs from the F_2_ of AIL (Sheng et al., 2013). Based on these data, we characterized the gradient of the population structure over these generations and the potential functional genes of growth traits by a genome wide association study (GWAS), selective sweep analysis, haplotype association, and ancestral inference. The integrated analysis of selection in F_0_ and GWAS for AIL provides both power and precision and demonstrates the transmission of important genetic information between generations.

## Materials and Methods

### The AIL Population

The High Quality chicken Line A (HQLA) is a closed population founded by the commercial Anak broiler breed and a Chinese indigenous chicken line, followed by strong artificial selection over more than 10 generations, according to a weight-based selection index, while maintaining the meat quality and plumage color. The Huiyang Bearded chicken (HB) is a Chinese meat-type breed with a long history (1,600 years), which is characterized by its slow growth, high meat quality, and muff and beard phenotype (Guo et al., 2016); currently, HB is in the stage of conservation and breeding. At 7 weeks of age, the HQLA was 3.2-times the body weight of HB (Figure 1). The F_2_ cross was generated by the reciprocal crossing of the founder lines [4 HQLA♂ × 12 HB♀ and 4 HB♂ × 12 HQLA♀, details presented in Sheng et al. (2013)]. Later AIL generations (F_3_ to F_9_) were founded by birds from the F_2_ population and bred using random mating (Figure 1). The population size of each generation was maintained at more than 1,000 individuals. The body weight at 7 weeks of age (BW7) was around 900 g.

### Phenotype

For the F_9_ generation, body weight was measured at hatching and every other week until 12 weeks of age. During weeks 4–12, the shank length and shank circumference were also measured every 2 weeks. Boxplots for each phenotype were generated to scan for outliers. Individuals that were further than 1.5 times IQR away from the upper or lower quartile of the boxplots were removed. Descriptive statistics of the phenotypes are provided in Supplementary Table S1.

### Genotype

We employed GBS SNPs of F_0_, F_8_, and F_9_ (Wang et al., 2017) and Illumina Chicken 60K Beadchip SNPs of F_2_ (Sheng et al., 2013) for further filtering and analysis. In brief, for F_9_ generation, double-enzyme GBS (*Eco*RI/*Mse*I) libraries were prepared and sequencing was performed on a Illumina Nextseq500 sequencer. The TASSEL-4.0 GBS analysis pipeline was used to discover SNPs. Using VCFtools (0.1.17), the raw GBS SNP filter criteria was set to: –maf 0.05 –max-alleles 2 –min-alleles 2 –minDP 5 –minGQ 98 –max-missing 0.2 (Danecek et al., 2011). Genotype phasing of the clean SNPs was done using Beagle 5.0 (Browning and Browning, 2007) with gt model and impute = true parameters, other parameters were left as default. The GBS SNPs were evenly distributed across chromosomes (Supplementary Figure S1). In F_2_ generation, SNPs (autosome 1–28) that failed to meet the following criteria were removed: individual call rate (>0.9), individual SNP call frequency (>0.9), and minor allele frequency (MAF > 0.05). All the genomic coordinates of the SNPs were uniformly converted to the chicken reference genome *Gallus gallus*, version 5.0 (Ensembl release 94). After that, we kept 161,376 GBS SNPs for 16 HQLA, 14 HB, 185 F_8_, 602 F_9_ individuals, and 43,472 Chip SNPs for 492 F_2_ individuals.

### Genetic Parameter Estimation

We evaluated the changes in the population genetic parameters as a component of generation transmission. LD decay statistics were analyzed by PopLDdecay 3.31 (Zhang et al., 2019) with a max distance of 2 Mb. The inbreeding coefficient (F), nucleotide diversity (π), nucleotide divergent, and minor allele frequency (MAF) were evaluated by VCFtools (0.1.17) (Danecek et al., 2011). The heritability and genetic correlations of all traits were estimated using GCTA package (v1.92) (Yang et al., 2011).

### Genome Wide Association Study

The mixed linear model (MLM) approach was used for the GWAS of the F_9_ generation, as implemented in the GCTA package (v1.92) (Yang et al., 2011). The basic model was: *y* = *a* + *bx* + *g* + *e*, where *y* is the phenotype, *a* is the mean term, *b* is the additive effect (fixed effect) of the candidate SNP to be tested for association, *x* is the SNP genotype indicator variable, *g* is the polygenic effect (as captured by the GRM calculated using all SNPs), and *e* is the residual. The GWAS statistical model of body weight included the sex and batch as discrete covariates and hatch weight as a quantitative covariate. For shank traits, body weight at the same age were also included as a covariate, because we focused on QTL scans which are associated with bone growth. A quantile-quantile (Q-Q) plot generated in CMplot^1^ was used to assess the potential impact of population stratification (Supplementary Figure S2). Bonferroni correction was applied to correct the number of estimated independent markers. A subset of SNPs that were in approximate linkage equilibrium was obtained by removing one in each pair of SNPs if the LD was greater than 0.2 using PLINK v1.07 (Purcell et al., 2007). QTL intervals were established after the remaining top SNPs and their neighboring SNPs with *r*^2^ >0.3.

### Selective Sweep

To investigate the signatures of selection between HQLA and HB, four statistical tests were used, including XP-EHH and iHH (linkage disequilibrium-based), Tajima’s D (frequency spectrum-based), and *F*_st_ (population differentiation-based), to investigate the signatures of selection between HQLA and HB. The XP-EHH and iHH value at each locus were estimated by The selfscan program (v1.2.0a) (Szpiech and Hernandez, 2014), and the genetic map for our population was 3 cM/Mb. The statistics for *F*_st_ and Tajima’s D were calculated using VCFtools (0.1.17) (Danecek et al., 2011) with a window size of 200 Kb and step size of 100 Kb.

### Haplotype-Based Association Analyses

A haplotype-based association analysis was performed in the ∼120 Kb fine-mapped QTL region on GGA27 using the following model:

***Y*** = ***X****β* + ***Z****u* +*e*

where **Y** is a column vector containing the SL10 of the F_9_ individuals. **X** is the design matrix including the coding for the sex of the birds. For each specific interval, there are n haplotypes for m individuals constructed by several SNPs. **Z** is the design matrix (m × n) containing each haplotype count (coded as 0, 1, 2) of each individual. β is a vector that estimates the fixed effect of sex, u is a column vector that estimates the allele substitution effects for each haplotype, and e is the normally distributed residual.

### Ancestral Inference

The RFmix software (v2.03) (Maples et al., 2013) is based on a discrimination analysis model that can be used to estimate the genetic ancestry composition of each individual and each chromosome. Using the F_0_ population as the ancestor population, RFmix was used to evaluate the local ancestral source of individuals in the F_9_ generation. To determine the haplotype window size, we set conditional random field spacing (# of SNPs) (-c) to 9 based on the results of LD with *r*^2^ = 0.2 as the critical value, and generations since admixture (-G) set to 9. Other parameters were left as default.

## Results

### Genetic Architecture of the AIL Population

**FIGURE 2:**
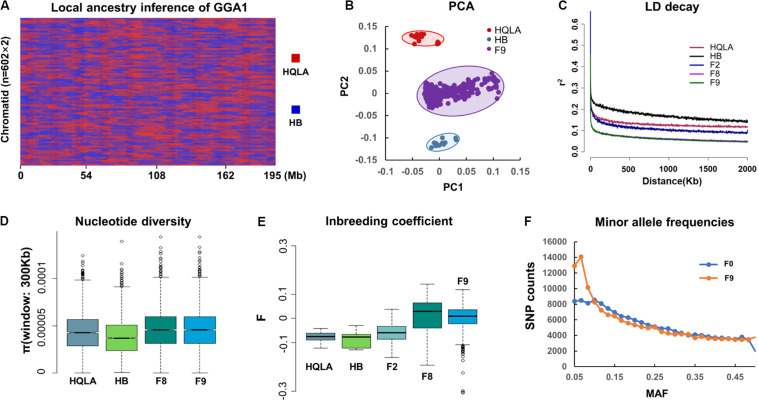
Genetic evaluation of the AIL population. **(A)** The HQLA-HB type recombination events accumulated from F_0_ to F_9_, taking chromosome 1 of all F_9_ sequencing samples (*n* = 602) as an example. Local ancestors are marked with HQLA in red and HB in blue. **(B)** PCA (principal component analysis). **(C)** The extent of the LD in different generations of AIL. Values are the mean LD r^2^ values for all pairs of SNPs binned by distance. The nucleotide diversity (300 Kb windows) and inbreeding coefficient are shown in **(D,E)**. **(F)** Minor allele frequency (MAF) distribution for the populations of F_0_ and F_9_.

Inspection of the 161 K variants segregating in AIL chicken identified several notable characteristics. The ancestral genome regions that inherited HQLA and HB were uniformly distributed and fully mixed in the F_9_ generation (Figure 2A). A total of 156,664 HQLA-HB type recombination events were identified on 1,204 chromosomes (602 individuals on GGA1 to GGA28). Each F_9_ produced an average of 260.24 ± 21.92 crosses, and the average ratio of HQLA and HB ancestral components was 51.9–48.1%. PCA showed that all F_9_ individuals were clustered in the middle of the two founders, and we did not detect a widespread population structure or cryptic relatedness in the F_9_ population (Figure 2B), which prevented false positive associations.

LD (r^2^) decays in HQLA were significantly faster than those in HB, which is consistent with the ancestral cross history of HQLA (Figure 2C). The F_2_ generation is characterized by limited short-range recombination and continued to accumulate as the distance increased. The r^2^ decays rapidly in F_8_ and F_9_ individuals in comparison to F_2_ populations (*r*^2^_0.1 =_ 27 Kb in F_9_ and *r*^2^_0.1 =_ 570 Kb in F_2_), supporting the suitability of the F_9_ population for high-resolution mapping.

We used GBS SNPs to estimate the nucleotide polymorphisms (π) in each population (except F_2_) (Figure 2D) and the inbreeding coefficient (F) (Figure 2E). HQLA showed higher nucleotide polymorphisms but lower heterozygosity levels (higher F value) than HB. This profile is consistent with the strong artificial selection history of the HQLA population. Fortunately, the F_9_ generation maintained a high nucleotide polymorphism, and only 552 SNPs (0.34%) were lost compared to the F_0_ GBS data. Considering the distribution of minor allele frequencies (MAFs) (Figure 2F), a high proportion SNPs (30.43%) in F_9_ had lower allele frequencies (MAF < 0.1) than F_0_ (22.46%). This pattern shows that the AIL population still experiences a slight genetic drift and bottleneck between F_0_ and F_9_.

### GWAS Identified Two Major QTLs Affecting Growth Traits in the F_9_ Generation

**FIGURE 3:**
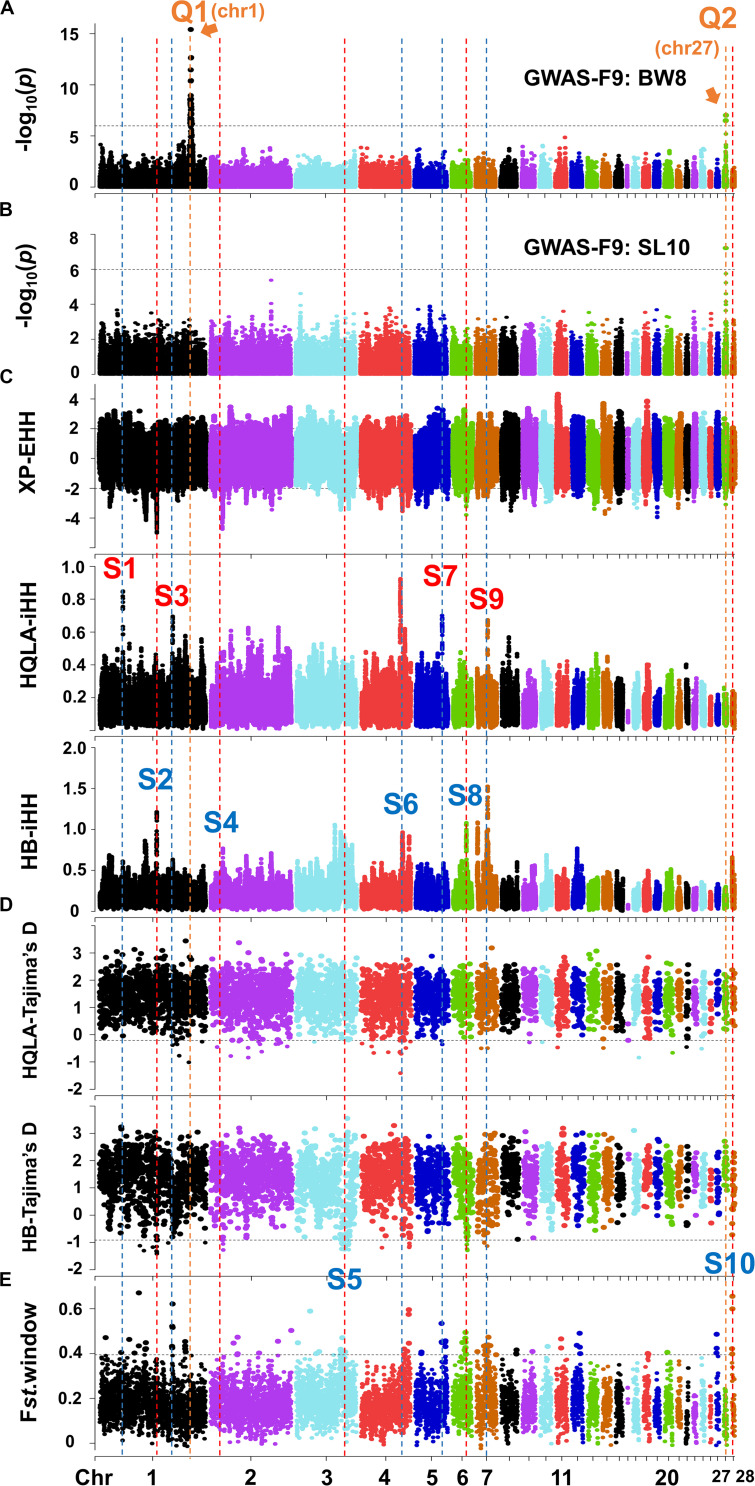
Joint analysis of GWAS in F_9_ and selection signature identification in F_0_. The Manhattan plots for BW8 **(A)** and SL10 **(B)**. The genome-wide 5% significance threshold -log_10_*P* was 5.99. **(C)** XP-EHH and iHH in HQLA and HB using a ±2 cutoff (top 4.4% genomic region). **(D)** Tajima’s D in HQLA and HB and **(E)** the F*_st_ value* with a 200 Kb window using the 99th percentile cutoff. The orange vertical dashed (marked by the letter Q) represents the QTL interval, and the red and blue vertical dashed (marked by the letter S) represent the selection signature intervals dominated by HQLA and HB, respectively.

The growth traits of this study population have high heritability (0.48–0.82, Supplementary Table S2). Using a mixed linear model, we performed GWAS between the 161,376 GBS SNPs and 17 growth traits, including BW2-BW14, SC4-SC12, and SL4-SL12, in 599 F_9_ individuals. All traits have a high phenotypic and genetic correlation, especially between the same traits at different periods. The correlation between SC and BW at different periods is higher than the correlation between SL and BW (Supplementary Table S3). At a Bonferroni of 5% (1.01 × 10^–6^, 0.05/49,318), we identified a large major QTL mainly affecting body weight at GGA1: 168.6–171.7 Mb (Q1) and a small major QTL mainly affecting shank development at GGA27: 3.60–3.72 Mb (Q2) (Figures 3A,B and Supplementary Figure S3). These QTL peaks were narrower than those of the F_2_ linkage analysis (Sheng et al., 2013). The most significant associations were for BW8 at GGA1: 169,241,142 bp (*p* = 3.8 × 10^–16^) and SL10 at GGA27: 3,608,297 bp (*p* = 6.1 × 10^–8^). Q1’s confidence interval was 25-fold that of Q2, partly because the recombination rate of GGA27 (12.05 cM/Mb) was 4.9-fold that of GGA1 (2.44 cM/Mb) (Sheng et al., 2013) and the LD in GGA1 is more extensive than that in GGA27 (Supplementary Figure S4). The broad loci in Q1 make it difficult to infer which genes are responsible for the association. We speculate that there is more complex genetic architecture concealed in Q1, such as multiple linked minor QTLs. However, clarifying this architecture further is a very difficult. The following fine-mapping work mainly focuses on the Q2 interval. It should be noted that the Z chromosome was excluded in this study due to the pre-GWAS of 297 cocks (ZZ) showing no significant signal on the Z chromosome.

### Selective Sweep Analysis on the F_0_ Generation Retrieved a Missing QTL on GGA4

The genes or variants underlying the large phenotypic differences between HQLA and HB likely evolved rapidly after artificial selection. Based on this principle, we employed different statistical tests to investigate the signatures of selection, including frequency spectrum-based Tajima’s D, the linkage disequilibrium-based XP-EHH method, and the population differentiation-based *F*_st_ method. However, one must carefully evaluate the results of selection signals since small sample sizes may introduce large drift effects. Hence, we combined our GWAS results with the Animal Quantitative Trait Loci Database^2^ to conduct a further screening of each selection signal interval. By comparing the growth traits associated QTLs with the candidate selection interval obtained by at least one method, we identified a total of 10 clear selection signal intervals (S1–S10), four of which occurred mainly in HQLA and six of which occurred in HB (Figures 3C–E).

**FIGURE 4:**
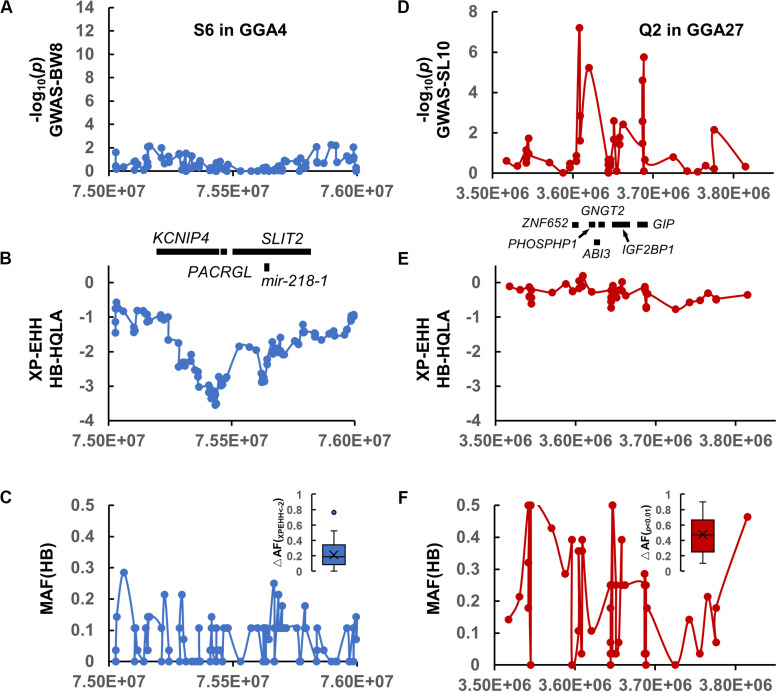
Comparison of the selection interval S6 in **(A–C)** and the QTL interval Q2 in **(D–F)**. **(A**,**D)** The association results of S6 on GGA4 and Q2 on GGA27 with the genes displayed below. **(B**,**E)** The XP-EHH signature of S6 and Q2. **(C**,**F)** The MAF distribution for HB and the allele-frequency differences between the HQLA and HB (ΔAF) in S6 and Q2, respectively.

Among these, we highlight the narrowed S6 interval on GGA4 (Figures 3C, 4A,B) matched the QTL database’s lists of growth traits. This signal spans GGA4: 75.28–75.67 Mb, harbors some candidate genes (*PACRGL*, *SLIT2*, *KCNIP4*, and *mir-218-1*), and has been reported to be significantly associated with chicken body weight in different populations, such as White Leghorn × Rhode Island Red cross (Sasaki et al., 2004), Silky Fowl × White Plymouth Rock cross (Gu et al., 2011), Beijing-You chickens (Liu et al., 2013), New Hampshire × White Leghorn cross (Nassar et al., 2015), and Dongxiang Blue-shelled chickens × White Leghorn cross (Guo et al., 2020). However, our association results were negative at this location because HQLA and HB were selected in the same direction (nearly fixed in HB, Figure 4C), resulting in an extremely low allele frequency difference (ΔAF) between them, which led to a further loss of statistical power in F_9_-GWAS.

### Fine-Mapping and Local Ancestral Inference for the Mosaic QTL on GGA27

A 120 Kb QTL region (GGA27: 3.60–3.72 Mb) was identified by 34 GBS SNPs and aggregated using *r*^2^ >0.3 with the top five SNPs of SL10. The GWAS significant SNPs were not continuously distributed across the region but were instead located in two peaks separated by regions with no genetic hitchhiking (Figure 4D). It is difficult to identify the selected interval by the window-based selection method under large allele frequency fluctuations (Figures 4E,F) (the mosaic association model).

**FIGURE 5:**
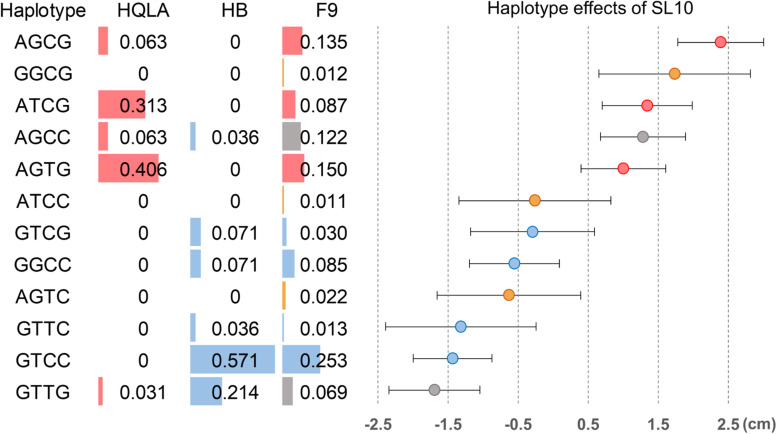
Haplotype association analysis for the shank length at 10 weeks of age in the 120 Kb candidate region on GGA27. Four SNPs were associated with SL10 in a multilocus backward-elimination analysis across the segment. Haplotypes were estimated in the founder populations (HB and HQLA) and the F_9_ AIL generation across these markers. Twelve haplotypes were inferred in F_9_ at haplotype frequencies (HF) >0.01, including five unrecombined HQLA haplotypes (red), six unrecombined HB haplotypes (blue), two shared haplotypes (gray), and three recombination haplotypes (orange). Overall, the haplotype substitution effects exhibited a gradual distribution of effects on SL10 in F_9_.

We further analyzed the genetic architecture of this QTL using haplotype association analysis. To identify the haplotypes contributing to the association signal, a multilocus backward-elimination analysis was performed across the 34 SNPs in the 1.2 Mb region, and the top SNP on GGA1:169,241,142 bp was selected to control for Q1 effects. Four SNPs (GGA27: 3,608,297 bp, 3,620,306 bp, 3,644,245 bp, and 3,686,628 bp) were identified to have statistically independent associations with SL10 at a 5% False Discovery Rate (FDR) threshold. The haplotypes tagged by these 4 SNPs were estimated; in total, 12 haplotypes were detected (MAF > 0.01 in F_9_). Tracing back to the F_0_ generation, we confirmed three HQLA-origin haplotypes (A, red), four HB-origin haplotypes (B, blue), two shared haplotypes (gray), and three recombination haplotypes (orange) (Figure 5).

We first focused on the dominance-recessiveness relationship and computed the phenotype scale for AA, AB, and BB as 1.37 ± 0.65 cm (*n* = 95), 0.08 ± 1.22 cm (*n* = 201), -1.53 ± 0.64 cm (*n* = 151), respectively. The results showed that HQLA carries the main length increasing alleles, and heterosis does not commonly exist in crosses of AB. Next, the additive haplotype substitution effects on SL10 were estimated. There was a gradient distribution of haplotype allele effects between decreasing SL10 by 1.57 cm and increasing it by 2.38 cm (Figure 5). Although most of the length increasing haplotypes are inherited from HQLA, there is still fluctuation among them, which is the same in HB. This effect distribution does not seem to be caused by only one causal mutation. Some well-known candidate genes related to body size and bone growth are located within this interval and are worthy of follow-up research, including *PHOSPHO1*, *IGF2BP1*, *ZNF652*, and *GIP* (Figure 4D). However, the proportion of our recombination haplotypes is too small to give further genetic evidence in the current population. Further recombinations (more offspring in AIL F_n_) and higher density markers (Davies et al., 2016; Yang et al., 2019) will clarify this issue.

## Discussion

Crosses among well-characterized strains are a mainstay of modeling organism genetics. We reported a running chicken AIL that was generated by crossing HQLA × HB, which was differentially bred for fast growth and slow growth prior to subsequent intercrossing. Systematic characterization of the genetic architecture of AIL makes it possible to evaluate the suitability of different genomic situations for GWAS. Overall, the F_9_ of AIL has low linkage disequilibrium between markers to obtain accurate mapping resolution, an absence of population structure to prevent false positive associations, and relatively stable allele frequency to ensure a high enough power to detect the majority of quantitative trait loci (QTLs). We highlighted the fine-mapped QTL on the GGA27 derived from GWAS, haplotype association, and local ancestry inference, which implicated four candidate genes corroborated by extant human, mouse, and chicken genetic data.

Although the basic strategy to build the AIL was similar to that in other studies, certain practical considerations, along with the AIL’s complex genetic background, affected the design of this study and its outcomes in important ways. For example, we observed rich diversity and intense LD decay in the F_0_ generation, even though the four males were full siblings, and the 12 females were either half or full siblings, in the HQLA and HB founders, which is very different from the inbred AIL line of mice (Gonzales et al., 2018) and other model organisms (Mackay et al., 2012; Nicod et al., 2016). Compared with other chicken AILs, HQLA-HB-AIL presents lower levels of SNP diversity loss (11% SNPs with MAF < 0.05 in F_9_) than broiler × Fayoumi AIL (60% SNPs with MAF < 0.05 in F_18_ and F_19_) (Van Goor et al., 2015). Moreover, our previous study (Guo et al., 2016) reported that the single-nucleotide genome-wide polymorphisms of F_0_ were 1.38-fold those of the founders of the Virginia chicken AIL population (Zan et al., 2019), which illustrates the high polymorphisms in our population. Thus, this AIL is more human-like or similar to laboratory outbred mice (Yalcin et al., 2010) than the inbred AIL mice model, namely that this AIL has lower levels of LD, lower MAFs, and is more abundant haplotype diversity; the resulting mosaic association model also supports this conclusion. This is a double-edged sword that improves fine-mapping accuracy but affects power by increasing the multiple testing burden (Parker et al., 2016). In addition, the diversity of F_0_ may be due to the breeding process because HQLA is a commercial strain formed first by crossing and then by directed selection. This factor gives this population greater similarities to the three-ancestor MASIC population from the perspective of ancestors, which can be monitored by estimating individual ancestry (Supplementary Figure S5) using the unsupervised ADMIXTURE method (Alexander et al., 2009).

We also presented a joint analysis of GWAS, and selective sweep of this AIL was able to comprehensively extract more genomic features. Firstly, although we cannot rule out the effect of genetic drift on the selection results, the diversity of F_0_ still reduces the false positive rate of the selection signal to some extent, which allows all candidate intervals to be further studied based on their association with other phenotypes. Secondly, we showed a typical example of failing to replicate prior results on GGA4, which we explained by the loss of GWAS power that results from rare alleles. This result demonstrated that local diversity may be lost, even if two founder strains generally maintain large phenotypic/genotypic differences, which is also a major performance difference between AIL and MAGIC. Besides, this study used ∼160 K SNPs, which means that a very large sample size which not available currently is required to meet the multiple testing correction of detecting epistatic QTLs. Therefore, a comprehensive analysis of interaction between directional epistasis and mutation effects will also be a very interesting issue to be explored in the near future.

Another core issue of this study is the dissection of key growth-related (especially for bone development) genes. We focused on the narrow QTL on GGA27 that contains fewer genes, and we highlighted some genes that are noted by the existing literature for their role in the corresponding traits. This result finely replicated the F_2_ finding (Sheng et al., 2013) and is consistent with the QTL-mapping in Japanese cockfighting (Tsudzuki et al., 2007) and Pekin ducks (Zhou et al., 2018). The lead SNP at chr27:3,608,297 is associated with the shank length in F_9_ AILs, which lies in the intron of the *ZNF652* gene. Although the function of this gene has not been reported in detail, discoveries from human GWAS have replicated the significant correlation between *ZNF652* and body height in two independent cohorts [rs35587648, *p* = 7 × 10^–42^ in Lango Allen et al. (2010) and rs2072153, *p* = 4 × 10^–8^ in Kichaev et al. (2019)]. Interestingly, three other genes at this locus, *PHOSPHO1*, *IGF2BP1*, and *GIP*, have been reported to be related to skeletal development. PHOSPHO1 is a phosphoethanolamine/phosphocholine phosphatase that has been implicated in the generation of Pi for matrix mineralization, a process central to skeletal development. *Phospho1*^–^*^/^*^–^ mice display growth plate abnormalities, spontaneous fractures, bowed long bones, osteomalacia, and scoliosis in early life. Insulin-like growth factor II mRNA-binding protein 1 (*IGF2BP1*) belongs to a family of RNA-binding proteins implicated in mRNA localization, turnover, and translational control (Bell et al., 2013). The *IGF2BP1*^–^*^/^*^–^ mice were, on average, 40% smaller than their wild-type and heterozygous littermates; growth retardation was apparent from E17.5 and remained permanent into adult life (Hansen et al., 2004). Moreover, a GWAS study revealed that a putative regulatory mutation causes the continuous expression of the *IGF2BP1* gene after birth, which increases body size of Pekin ducks by 15% (Zhou et al., 2018). Glucose-dependent insulinotropic polypeptide (*GIP*) also has been recognized in the last decade as an important contributor to bone remodeling and is necessary for optimal bone quality (Guo et al., 2020). GIP stimulates osteoblasts and increases bone formation. A decline in GIP leads to a decline in bone metabolism, which could be one of the mechanisms that induces osteopenia in diabetics (Zofkova, 2015). It is possible that all four genes are associated with shank length and further affect body weight as they are all involved in growth traits, which is also suggested by the progressive haplotype accumulation effect (Figure 5). In short, the above genes provide a starting point to further study the shank traits. The next analysis requires multi-omics methods, i.e., combined with a map-based approach, gene expression analysis, metabolic regulation analysis, causality analysis, and other optional methods to investigate the molecular mechanism and causal mutations in this region.

In summary, the HQLA-HB-AIL chicken, which balanced the avoidance of rare alleles with the requirement for rapid linkage disequilibrium (LD) decay, is a reasonable resource for detecting quantitative trait genes. This AIL yielded a much narrower QTL than the F_2_ generations, especially the QTL on chromosome 27. Further, we highlighted the important role of four candidate genes (*PHOSPHO1*, *IGF2BP1*, *ZNF652*, and *GIP*) for bone development. We also identified a missing QTL on chromosome 4 via the joint analysis of GWAS and a selection signature analysis, which demonstrated the local limitations of this population but can be remedied by a multidimensional analysis. Overall, our study provides a promising resource for this field of study and will facilitate our understanding of the genetic mechanisms underlying chicken bone growth.

## Data Availability Statement

All data are published and can be found in Sheng et al. (2013) and Wang et al. (2017). The genotyping data can be found in doi: 10.6084/m9.figshare.12581063.v1. The phenotype data can be found in Supplementary Table 4.

## Ethics Statement

The animal welfare committee of the State Key Laboratory approved all animal care and experimental procedures for Agrobiotechnology of China Agricultural University with approval number SKLAB-2014-06-07.

## Author Contributions

XH, CL, HQ, NL, and YW conceived and designed the experiments. YW and XC performed the experiments. YW, LB, and CZ analyzed the data. YW, LB, XC, JR, and ZH contributed reagents, materials, and analysis tools. YW wrote the manuscript. DS, HQ, CL, XC, and XH provided comments on the manuscript. All authors contributed to the article and approved the submitted version.

## Conflict of Interest

The authors declare that the research was conducted in the absence of any commercial or financial relationships that could be construed as a potential conflict of interest.

## Footnotes

1 https://github.com/YinLiLin/R-CMplot

2 https://www.animalgenome.org/cgi-bin/QTLdb/index

## Supplementary Material

FIGURE S1 Chromosome-wise SNP density in the GBS panel in physical distance.

FIGURE S2 Quantile-quantile (Q-Q) plot of all 17 traits.

FIGURE S3 The Manhattan plots for 15 traits. Phenotypes including BW2 to BW6, BW10 to BW14, SC4 to SC12, SL4 to SL8, and SL12. The genome-wide 5% significance threshold -log_10_*P* was 5.99.

FIGURE S4 LD decay near two QTL interval. The red line represents the LD pattern of GGA1: 168–171 Mb (543 SNPs) and the black line represents the LD pattern of GGA27: 2.16–5.16 Mb (391 SNPs).

FIGURE S5 Analysis of population structure of F_0_ and F_9_. Supervised analysis showed that all F_9_ individuals were clustered in the middle of the two founders. Unsupervised analysis showed *K* = 3 is the best model that is consistent with the breeding process of HQLA and F_9_ cross.

TABLE S1 Descriptive statistics of the phenotypes.

TABLE S2 Heritability of 17 traits.

TABLE S3 Genetic and phenotypic correlation coefficient of 17 traits.

TABLE S4 The phenotype data.
