# Supplementary material for: Genetic Dissection of Growth Traits in a Unique Chicken Advanced Intercross Line
Source: Front Genet. 2020 Sep 4;11:894. doi: 10.3389/fgene.2020.00894 (PMC7509424; doi:10.3389/fgene.2020.00894)
Supplement: FIGURE S1 — Chromosome-wise SNP density in the GBS panel in physical distance. [file Data_Sheet_1.docx]

**Genetic Dissection of Growth Traits in a Unique Chicken Advanced Intercross Line**

Yuzhe Wang*^1,2†^*, Lina Bu*^2†^*, Xuemin Cao*^2^*, Hao Qu*^3^*, Chunyuan Zhang*^2^*, Jiangli Ren*^2^*, Zhuolin Huang*^2^*, Yiqiang Zhao*^2^*, Chenglong Luo*^3^*^*^, Xiaoxiang Hu*^2^*^*^, Dingming Shu*^3^*, Ning Li*^2^*

1. College of Animal Science and Technology, China Agricultural University, Beijing 100193, China

2. State Key Laboratory of Agrobiotechnology, College of Biological Sciences, China Agricultural University, Beijing 100193, China

3. State Key Laboratory of Livestock and Poultry Breeding, Guangdong Key Laboratory of Animal Breeding and Nutrition, Institute of Animal Science, Guangdong Academy of Agricultural Sciences, Guangzhou 510640, China

† These authors have contributed equally to this work.

* Corresponding author:

Xiaoxiang Hu, Email: [huxx@cau.edu.cn](mailto:huxx@cau.edu.cn).

Chenglong Luo, Email: [chenglongluo1981@163.com](mailto:chenglongluo1981@163.com)

**Supplementary Figure**

**
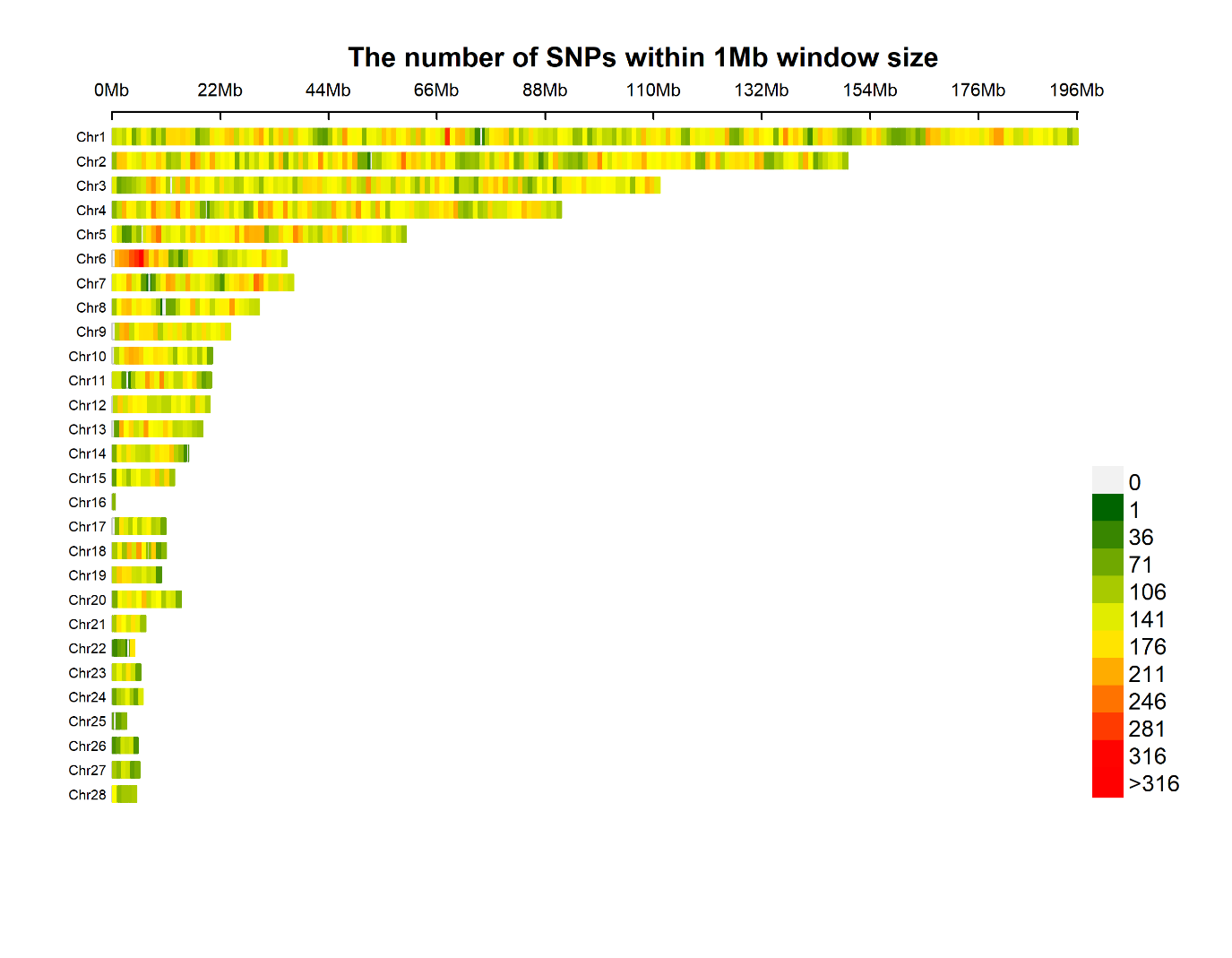
**

**Supplementary Figure 1 Chromosome-wise SNP density in the GBS panel in physical distance**


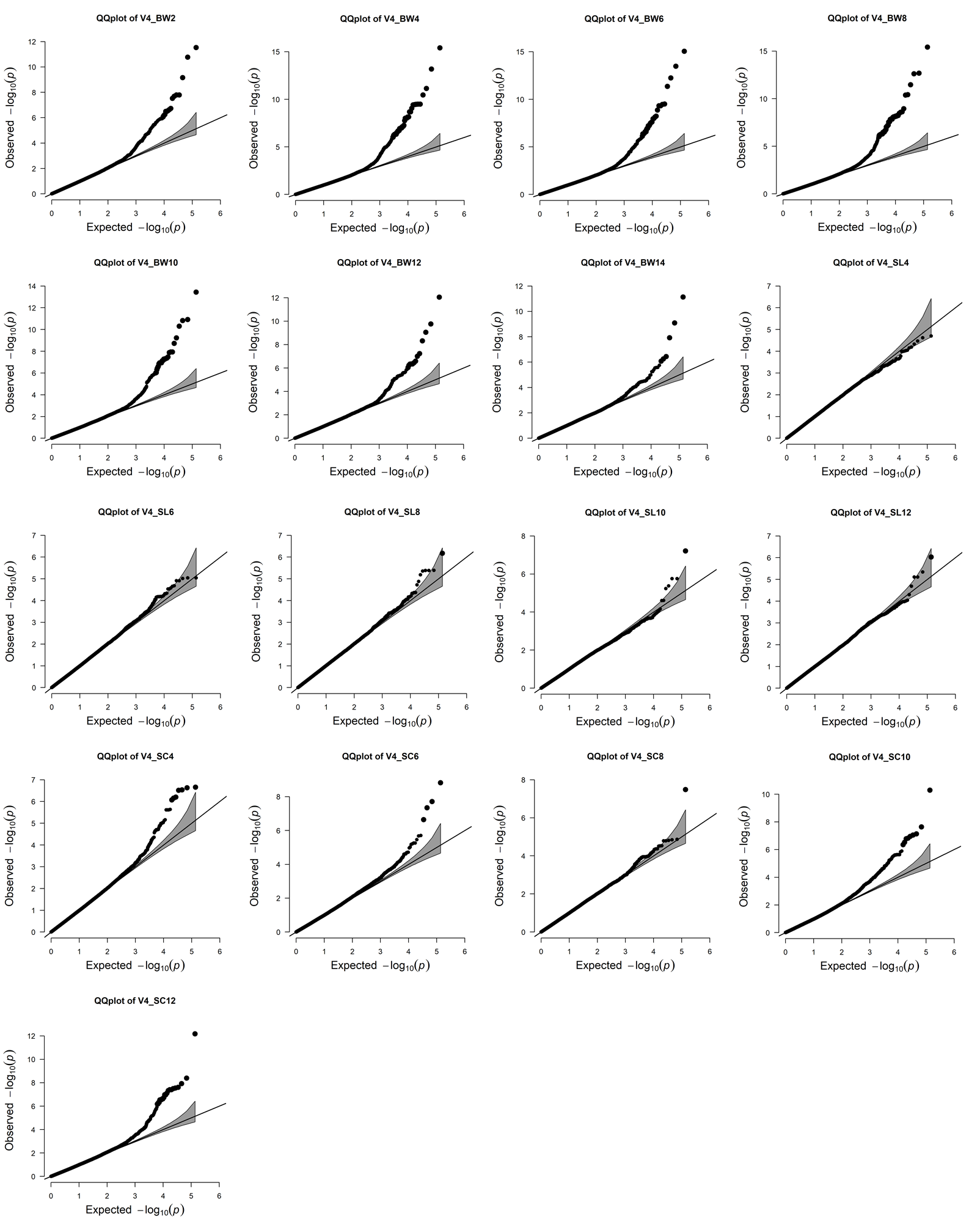


**Supplementary Figure 2 Quantile-quantile (Q-Q) plot of all 17 traits**

**
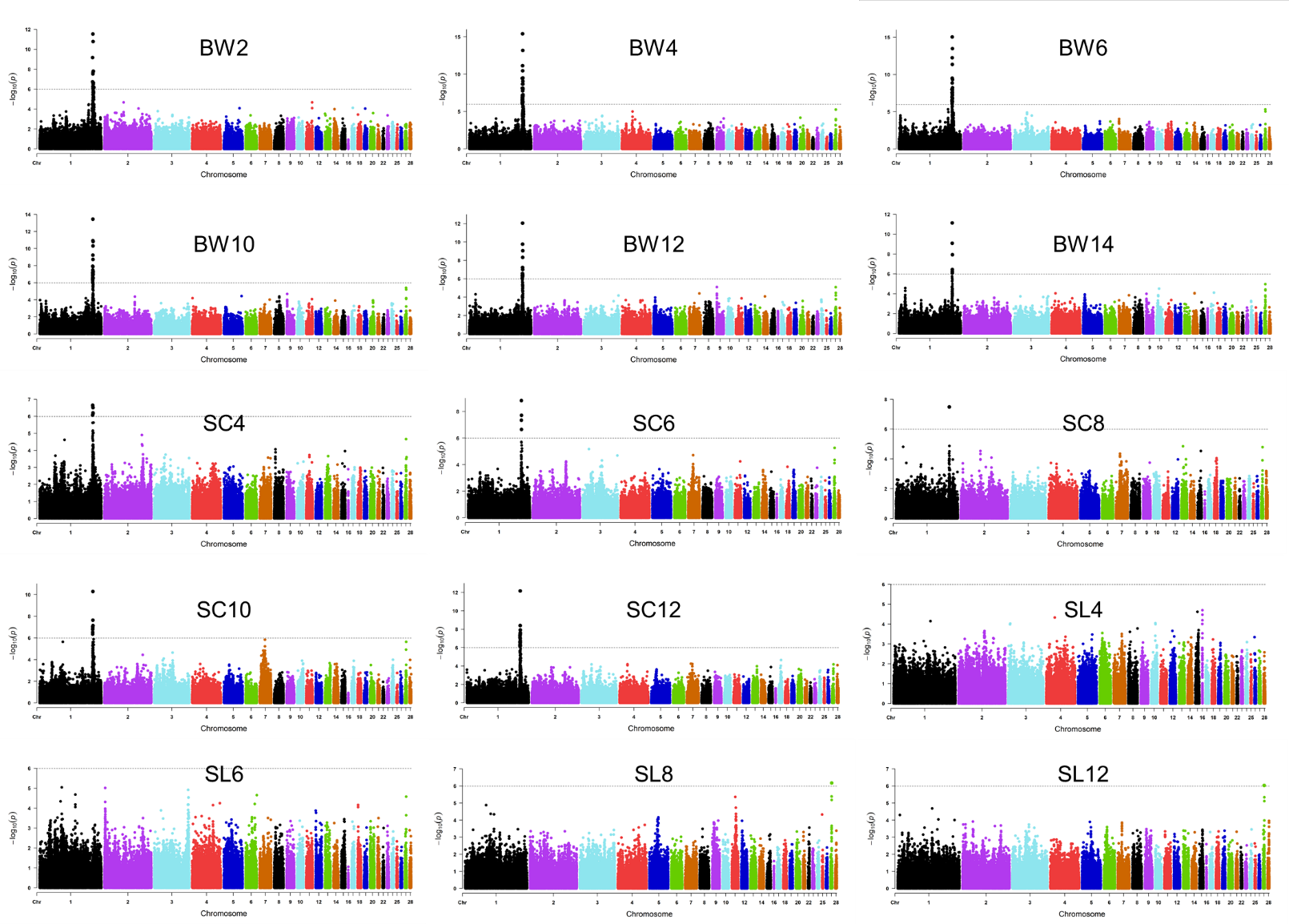
**

**Supplementary Figure 3 The Manhattan plots for 15 traits.** Phenotypes including BW2 to BW6, BW10 to BW14, SC4 to SC12, SL4 to SL8 and SL12. The genome-wide 5% significance threshold -log_10_*P* was 5.99.

**
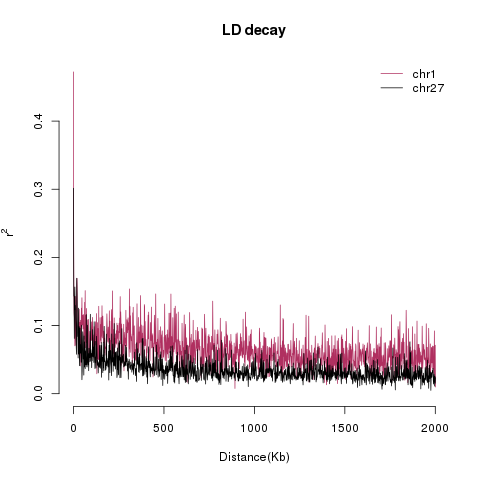
**

**Supplementary Figure 4 LD decay near two QTL interval.** The red line represents the LD pattern of GGA1: 168-171 Mb (543 SNPs) and the black line represents the LD pattern of GGA27: 2.16-5.16 Mb (391 SNPs).


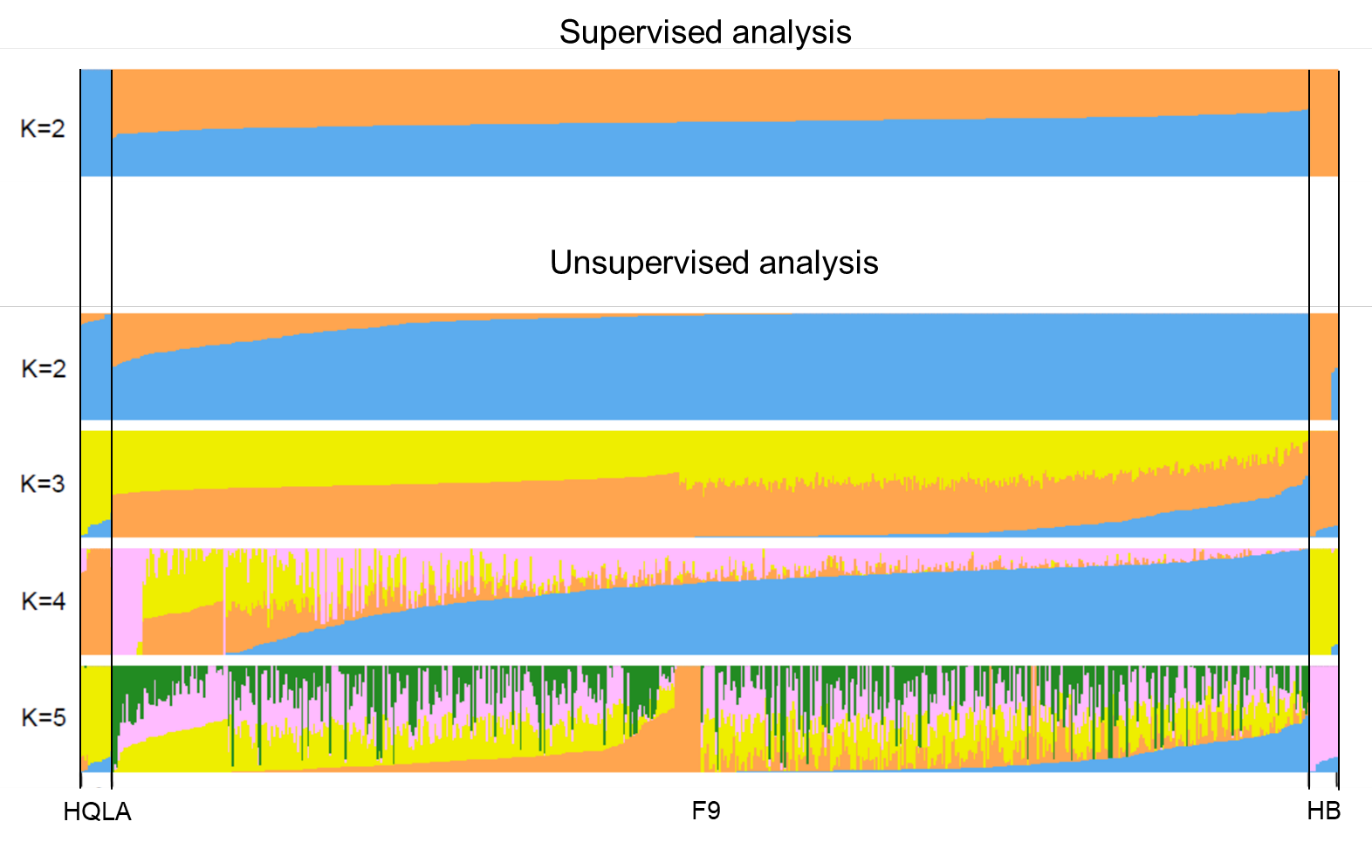


**Supplementary Figure 5 Analysis of population structure of F_0_ and F_9_.** Supervised analysis showed that all F_9_ individuals were clustered in the middle of the two founders. Unsupervised analysis showed K=3 is the best model that consistent with the breeding process of HQLA and F_9_ cross.
